# Significance of serological markers in the disease course of ulcerative colitis in a prospective clinical cohort of patients

**DOI:** 10.1371/journal.pone.0194166

**Published:** 2018-03-28

**Authors:** Gyorgy Kovacs, Nora Sipeki, Boglarka Suga, Tamas Tornai, Kai Fechner, Gary L. Norman, Zakera Shums, Peter Antal-Szalmas, Maria Papp

**Affiliations:** 1 Division of Gastroenterology, Department of Internal Medicine, Faculty of Medicine, University of Debrecen, Debrecen, Hungary; 2 Institute of Experimental Immunology, Euroimmun AG, Lübeck, Germany; 3 Inova Diagnostics, Inc., San Diego, California, United Statesof America; 4 Department of Laboratory Medicine, Faculty of Medicine, University of Debrecen, Debrecen, Hungary; Rambam Health Care Campus, ISRAEL

## Abstract

**Background & aims:**

To determine the prognostic potential of classic and novel serologic antibodies regarding unfavorable disease course in a prospective ulcerative colitis (UC) patient cohort, since few and conflicting data are available in the literature regarding this matter.

**Methods:**

187 consecutive patients were studied prospectively (median follow-up: 135 months) from a single referral IBD center in Hungary. Sera were tested for different IgA/IgG type autoantibodies (anti-neutrophil cytoplasmic [ANCA], anti-DNA-bound-lactoferrin [anti-LFS], anti-goblet cell [anti-GAB] and anti-pancreatic [PAB: anti-CUZD1 and anti-GP2)]) by indirect immunofluorescence technique and for anti-microbial (anti-*Saccharomyces cerevisiae* [ASCA] IgG/IgA and anti-OMP Plus^™^ IgA) antibodies by enzyme-linked immunosorbent assays.

**Results:**

A total of 73.6%, 62.4% and 11.2% of UC patients were positive for IgA/IgG type of atypical perinuclear-ANCA, anti-LFS and anti-GAB, respectively. Occurrences of PABs were 9.6%, while ASCA IgA/IgG and anti-OMP IgA were 17.6% and 19.8%, respectively. Antibody status was stable over time. IgA type PABs were more prevalent in patients with primary sclerosing cholangitis (37.5% vs. 4.7% for anti-CUZD1 and 12.5% vs. 0% for anti-GP2, *p<0*.*001* for both). IgA type ASCA and anti-CUZD1 antibodies were associated with higher risk of requirement for long-term immunosuppressant therapy in Kaplan-Meier analysis (pLogRank *<0*.*01* for both). However, in multivariate Cox-regression analysis only ASCA IgA (HR: 2.74, 95%CI: 1.46–5.14, *p<0*.*01*) remained independent predictor. UC-related hospitalization due to disease activity was only associated with multiple antibody positivity (for 3 or more; HR 2.03 [95% CI: 1.16–3.56]; p = 0.013). None of the individual antibodies or their combination was associated with the risk of development of extensive disease or colectomy.

**Conclusion:**

Even with low prevalence rates, present study gives further evidence to the role of certain antibodies as markers for distinct phenotype and disease outcome in UC. Considering the result of the multivariate analysis the novel antibodies investigated do not seem to be associated with poor clinical outcome in UC, only a classic antibody, IgA subtype ASCA remained an independent predictor of long-term immunosuppressive therapy.

## Introduction

Enhanced antibody formation in the serum is a well-known feature of inflammatory bowel diseases (IBD). A wide range of anti-microbial and autoantibodies have been reported to be associated with either Crohn’s disease (CD) or ulcerative colitis (UC) [1]. Anti-microbial antibodies are formed against different surface carbohydrate (glycans [2]) or protein antigens of various gut microbes [3], while autoantibodies are directed against host proteins. Currently, the most relevant anti-microbial antibody is the ASCA (anti-*Saccharomyces cerevisiae* antibody), while the major autoantibody is the ANCA (anti-neutrophil cytoplasmic antibody). The panel of serologic antibodies, however, has continuously been expanding [1] calling for clarification of whether these new markers are superior or add value to the conventional markers. Existence of serologic markers might be considered as a reflection of the enhanced microbial challenge to the gut [4, 5] due to a disturbed gut innate immune system that triggers an exaggerated adaptive immune response. These serologic antibodies may also be actively involved in the pathophysiology of gut inflammation in IBD [6, 7].

Serologic antibodies play a potential role in providing an insight into the etiopathogenesis of IBD, establishing the diagnosis of IBD and to differentiating CD from UC. Currently, their most fascinating and relevant potential is to stratify the risk of evolving a complicated disease course that might dictate earlier more aggressive treatment [8]. This latter issue was extensively studied in CD [1], however, data are few and conflicting regarding the association of serologic markers to the disease course [9–14], medical treatment and response to therapy [9, 10, 13] in patients with UC, especially with the newly discovered antibodies. Thus a comprehensive evaluation of a panel of serologic antibodies in a large prospectively followed UC cohort is required.

The aims of the present study were to investigate: (1) long-term stability of a panel of serologic antibodies comprising classic and newly discovered markers, (2) associations between the presence of antibodies and the clinical phenotype of the disease, (3) prognostic potential of these antibodies with regards to the long-term disease course in a large prospective referral adult UC cohort.

## Materials and methods

### Patient population

We performed a cohort study among adult UC patients in a Hungarian tertiary IBD referral center (Gastroenterology Department of Institute of Medicine, University of Debrecen). The baseline clinical data regarding this cohort overlap with our previous studies [15, 16], however hereby we present an extended follow up time with nearly 2 and a half years and re-evaluation of the outcomes. We used the same step by step thorough statistical evaluations; therefore the text appeared to reproduce information already reported in detail elsewhere.

Diagnosis of IBD was based on the Lennard–Jones criteria [17]. Detailed clinical phenotypes were captured at inclusion. Clinical data were determined by thorough review of patients’ medical records, which had been collected in a uniform format described in detail in our previous studies [15, 16]. Medical records that documented age at presentation, disease extent [18], presence of extraintestinal manifestations [EIM] and familial IBD, smoking habits, medication use, UC-related hospitalization due to disease activity, development of extensive disease (from E1/E2 to E3) and need for colectomy, were retrospectively analyzed for the period prior to the prospective follow-up. At enrolment, clinical disease activity was calculated according to the partial Mayo score [19]. Mayo score ≤ 3 was defined as a state of remission and >4 as a state of active disease. Endoscopic activity was determined according to the endoscopic component of the Mayo score [20]. A state of active disease was defined as ≥1 points according to endoscopic partial Mayo score.

### Phenotypical characterization of IBD patients during prospective follow-up

183 of 187 UC patients were available to be enrolled into a prospective follow-up study, where the treating IBD physicians registered laboratory data, endoscopic and imaging findings, disease activity, medical treatment, date of UC-related hospitalization, development of extensive disease (from E1/E2 to E3) and colectomy during regular and extraordinary outpatient follow-up visits and inpatient stays. Maximal disease extent (proctitis, left-sided colitis, and extensive colitis) [18], observed during endoscopic follow-up was also registered. UC-related hospitalization was defined as any admission for the treatment of UC disease activity. Colectomy performed for medically refractory disease was considered in analyses. In Hungary, a follow-up visit is usually scheduled for every 6 months at a specialized gastroenterology center (the actual interval varies between 3–6 months). The treatment algorithms, both the medical and the surgical, are harmonized and followed the ECCO guidelines. Need for colectomy and its timing is a consistent multidisciplinary decision with the collaboration of the gastroenterologist, radiologist, and surgeon [21–23]. Collected data were transferred and stored in a database for analysis. In May 31, 2015, all patients’ charts and database were reviewed and updated for the data points mentioned above. Follow-up for a particular patient was terminated if there was no further record available.

The treating physicians were aware of the antibody seropositivity of the patients, but did not incorporate them into their regular clinical decision making (e.g. treatment choices), except only in case of selected differential diagnostic problems (using ASCA and pANCA status to distinguish CD, especially patients with only colonic localization [L2 according to Montreal classification], from UC), alongside with ECCO guidelines on this matter [1, 24, 25].

### Serologic antibody determination

Sera obtained at enrolment were separated from venous whole blood and stored at -80°C.

Atypical P-ANCA, anti-LFS, anti-goblet, anti-GP2 and anti-CUZD1 IgA and IgG were detected using cell-based indirect immunofluorescence tests (IIFT) [Morbus-Crohn Mosaic 1, Euroimmun Medizinische Labordiagnostika AG, Lübeck, Germany] in a manner previously described [15]. A specific fluorescence at a dilution of 1:32 or higher was considered positive for P-ANCA and anti-LFS and 1:10 or higher for anti-goblet, anti-CUZD1 and anti- GP2 antibodies. The interpretation of ANCA pattern was based on the behavior of the specimens on ethanol- and formalin- fixed slides according to previously reported [26].

Both serum IgG and IgA levels of anti-*Saccharomyces cerevisiae* antibodies (ASCA) and anti-*OMP Plus*^*™*^antibodies were evaluated by enzyme-linked immunosorbent assay (ELISA) separately [QUANTA Lite^®^, Inova Diagnostics, San Diego, CA]. The results are presented as arbitrary units with a cut-off value for positivity of 25 Units.

Sera were documented both, in absolute values and in frequency of positivity. Additionally, in case of each antibody, a highest quartile was defined by titers above laboratory cut-off values belonging to the Q3-Q4 range (75^th^-100^th^ percentiles). We used these in the quantitative analysis of associations between antibody titers and poor disease outcomes.

To evaluate the stability of various serologic antibodies [status of positive or negative for a respective antibody], we analyzed samples from the same patient over various arbitrary time-points during the disease course. At least two serum samples were taken from each of the majority of UC patients [*n* = 106] and re-tested for all different serologic antibodies.

### Statistical analysis

Variables were tested for normality using Shapiro Wilk’s W test. Continuous variables were summarized as means (standard deviation [SD]) or as medians (interquartile range [IQR]) according to their homogeneity. To evaluate differences within patient subgroups, the following statistical methods were used. Categorical variables were compared with Fisher’s exact test or χ2 test with Yates correction, linear-by-linear association, as appropriate. Continuous variables were compared with Student’s t test, one-way analysis of variance [ANOVA], or Mann-Whitney’s U test or Kruskal-Wallis H test with *post hoc* analysis [Dunn’s multiple comparison test]. Kaplan-Meier survival curves were plotted for analyzing the association between categorical clinical variables or serologic antibodies and unfavorable disease outcomes during follow-up with LogRank testing or Cox-regression analysis in the time-dependent models. Associations are given as odds ratio [OR] and hazard ratio [HR] with a 95% confidence intervals [CI]. A 2-sided probability value < 0.05 was considered to be statistically significant. A post-hoc power analysis was performed in Stata (v13.0) with a detailed description of the evaluation and results provided in the Supplementary Material (S1 File). For statistical analysis, GraphPad Prism 6 [San Diego, CA] and SPSS 22.0 [SPSS, Chicago, IL], Stata (v13.0) [StataCorp. 2013. Stata Statistical Software: Release 13. College Station, TX: StataCorp LP] programs were used.

### Ethical considerations

The regional (the Institutional Review Board of the University of Debrecen) and national (the Hungarian National Review Board) committee (DEOEC RKEB/IKEB 3515–2011, 3880/2012/EKU [59/PI/2012]) for research ethics approved the study protocol. Each patient was informed of the nature of the study and signed an informed consent form.

## Results

### Clinical characteristics of UC patients

In all, 187 well-characterized, unrelated, consecutive UC patients with a complete clinical follow-up (age range at presentation: 8–68 years, at first sampling: 17–85 years) seen at our Outpatient Clinic were enrolled between January 1, 2005 and June 1, 2010. The clinical characteristics of the patients at time of inclusion and sample procurement are presented in Table 1. Median follow-up from the diagnosis months 135 [IQR]: 84–213.

**Table 1:** Clinical characteristics of patients with ulcerative colitis (UC).

	UC (n = 187)
**Male/female (n)**	86/101
Age at presentation (years)*	33 (23–43)
**Familial IBD**^1^	6 (3.2%)
**Disease extent at diagnosis**^1^	
**E1**	30 (16.0%)
**E2**	104 (55.6%)
**E3**	53 (28.3%)
**Primary sclerosing cholangitis**	8 (4.3%)
**Extraintestinal manifestations (EIM)**	
**Arthritis**	26 (13.9%)
**Skin**	16 (8.6%)
**Ocular**	12 (6.4%)
**Smoking habits**^1^	
**never**	167 (89.3%)
**yes**	18 (9.6%)
**past**	2 (1.1%)
**Disease activity at study enrollment**^1^	
**Inactive partial Mayo ≤ 3**	135 (72.2%)
**Active partial Mayo**> **4**	52 (27.8%)
**Follow-up (months) from**	
diagnosis*^☐^	135 (84–213)
**sampling**	78 (51–102)
**Maximal disease extent**^1^^,^^🛇^	
**E1**	23 (12.8%)
**E2**	97 (53.9%)
**E3**	60 (33.3%)
**UC related hospitalization**^1^	64 (35.0%)
**Exposure of medication and surgery during follow-up**	
**Steroid use**^1^	117 (63.9%)
**Steroid refractory**^1^	11 (7.6%)
**Azathioprine use**^1^	70 (38.3%)
**Biological use**^1^	25 (13.4%)
**Colectomy**^1^	11 (6.0%)

^1^n (%),

*: median (IQR)

^**☐**^: 183 UC patients had follow-up from the diagnosis

^🛇^180 data were available

Disease extent: E1: proctitis, E2: left-sided colitis, E3: extensive colitis

### Frequency of serologic antibodies

A total of 73.6%, 62.4% and 11.2% of UC patients were positive for IgA/IgG type of atypical P-ANCA, anti-LFS and anti-GAB, respectively. Both types of PAB occurred as well, 9% of the patients were positive for anti-CUZD1 (≈ anti-rPAg1) and 0.6% for anti-GP2 (≈ anti-rPAg2) IgA/IgG. ASCA IgA/IgG and anti-OMP IgA positivity was 17.6% and 19.8%, respectively. Frequencies of the different antibodies in UC patients are summarized in Table 2.

**Table 2:** Serologic antibodies in patients with ulcerative colitis (UC).

Serologic antibodies	Type	Positive Cut-off	N	UC, n(%)
***Atypical P-ANCA***	***Either***	***1*:*32***	***178***	***131 (73*.*6%)***
	IgG			125 (70.2%)
	IgA			72 (40.4%)
***Anti-LFS***	***Either***	***1*:*32***	***178***	***111 (62*.*4%)***
	IgG			111 (62.4%)
	IgA			27 (15.2%)
***Anti-goblet cell***	***Either***	***1*:*10***	***178***	***20 (11*.*2%)***
	IgG			11 (6.2%)
	IgA			11 (6.2%)
***Anti-CUZD1*** ***(≈ rPAg1)***	***Either***	***1*:*10***	***178***	***16 (9*.*0%)***
	IgG			12 (6.7%)
	IgA			11 (6.2%)
***Anti-GP2*** ***(≈ rPAg2)***	***Either***	***1*:*10***	***178***	***1 (0*.*6%)***
	IgG			0 (0.0%)
	IgA			1 (0.6%)
***ASCA***	***Either***	***25 U/ml***	***187***	***33 (17*.*6%)***
	IgG			21 (11.2%)
	IgA			21 (11.2%)
***Anti-OMP***	***IgA***	***25 U/ml***	***182***	***36 (19*.*8%)***

ASCA: anti-*Saccharomyces cerevisiae* antibody, LFS: lactoferrin, CUZD1: CUB and zona pellucida-like domains 1,GP2: glycoprotein 2, P-ANCA: perinuclear anti-neutrophil cytoplasmic antibodies

### Stability of serologic antibodies

Median time between sample procurements was 21.1 months [IQR, 11.2–41.1]. Interestingly, the status of most serologic antibodies was very stable over time regarding both IgA and IgG subtypes, with only ≤ 10% of cases changing their antibody status over time. Atypical P-ANCA and anti-LFS antibodies, showed somewhat higher variation up to 23% of cases. Stability data of various serologic antibodies are summarized in Table 3. In case of anti-OMP IgA data regarding stability was available in only 23 UC patients, 82.6% of them were stable negative, while 17.4% appeared to be stable positive. None of them changed their antibody status over time.

**Table 3:** Stability of serologic marker status over time in patients with ulcerative colitis (UC) during the disease course.

Serologic antibodies	Type	N	Stable negative, n(%)	Stable positive, n(%)	Negative to Positive, n(%)	Positive to Negative, n(%)
**Atypical P-ANCA**	IgG	104	19 (18.3)	70 (67.3)	10 (9.6)	5 (4.8)
	IgA	104	46 (44.2)	34 (32.7)	9 (8.7)	15 (14.4)
**Anti-LFS**	IgG	104	26 (25.0)	54 (51.9)	14 (13.5)	10 (9.6)
	IgA	104	81 (77.9)	9 (8.7)	8 (7.7)	6 (5.8)
**Anti-goblet cell**	IgG	103	94 (91.3)	4 (3.9)	1 (1.0)	4 (3.9)
	IgA	103	93 (93.3)	6 (5.8)	0 (0.0)	4 (3.9)
**Anti-CUZD1 (≈ rPAg1)**	IgG	104	96 (92.3)	5 (4.8)	0 (0.0)	3 (2.9)
	IgA	104	95 (91.3)	2 (1.9)	2 (1.9)	5 (4.8)
**Anti-GP2 (≈ rPAg2)**	IgG	104	103 (99.0)	0 (0.0)	1 (1.0)	0 (0.0)
	IgA	104	102 (98.1)	0 (0.0)	2 (1.9)	0 (0.0)
**ASCA**	IgG	106	83 (78.3)	11 (10.4)	11 (10.4)	1 (0.9)
	IgA	106	86 (81.2)	9 (8.5)	8 (7.5)	3 (2.8)

ASCA: anti-*Saccharomyces cerevisiae* antibody, LFS: lactoferrin, CUZD1: CUB and zona pellucida-like domains 1,GP2: glycoprotein 2, P-ANCA: perinuclear anti-neutrophil cytoplasmic antibodies

In addition, no association was detected between the status of various serologic antibodies and the clinical or endoscopic disease activity [actual partial or endoscopic part of Mayo] at the time of sample procurement (data available in the public repository “Figshare” with the following related doi number: 10.6084/m9.figshare.4765102).

### Associations of serologic antibody profiles to clinical phenotype of the disease

No significant association was demonstrated between presence of serologic antibodies and gender, younger age at diagnosis (age ≤ 16 years), or colitis extent.

Presence of certain antibodies was less prevalent in patients with EIM: anti-LFS antibodies in ocular diseases (20.0% vs. 64.9%, *p = 0*.*004* for IgG subtype), while atypical P-ANCA (45.8% vs. 74.0%, *p = 0*.*005*for IgG subtype) and anti-LFS antibodies (0.0% vs. 17.5%, *p = 0*.*026* for IgA subtype) in arthritis. While other antibodies were more prevalent in patients with EIM: such as GAB in ocular diseases (40.0% vs. 9.5%, *p = 0*.*016*for IgG/IgA subtype). None of the antibodies was, however, associated with cutaneous manifestation of the disease.

IgA but not IgG types PABs were more prevalent in patients with PSC (37.5% vs. 4.7% for anti-CUZD1 and 12.5% vs. 0% for anti-GP2, *p<0*.*001* for both).

Lastly, presence of anti-LFS antibodies was negatively associated with current smoking status (No vs. Yes, 65.6% vs. 33.3%, *p = 0*.*01* for IgA/IgG subtype) as well.

All of these data are presented in Table 4.

**Table 4:** Associations between serologic antibody reactivities to different disease characteristics in patients with ulcerative colitis (UC).

		Atypical P-ANCA			Anti-LFS			Anti-Goblet			Anti-CUZD1 (≈ rPAg1)			Anti-GP2 (≈ rPAg2)			ASCA			Anti-OMP
EIMs		IgG	IgA	IgA and/ or IgG	IgG	IgA	IgA and/ or IgG	IgG	IgA	IgA and/ or IgG	IgG	IgA	IgA and/ or IgG	IgG	IgA	IgA and/ or IgG	IgG	IgA	IgA and/ or IgG	IgA
**Arthritis**	*p*	*0*.*005*		*0*.*02*		*0*.*026*												0.051	0.053	
	*OR [95% CI]*	*0*.*30 [0*.*12–0*.*72]*		*0*.*36 [0*.*15–0*.*87]*															0.17 [0.02–1.29]	
**Ocular**	*p*				*0*.*004*		*0*.*004*			**0.016**										
	*OR [95% CI]*				*0*.*14 [0*.*03–0*.*66]*		*0*.*14 [0*.*03–0*.*63]*			**6.33 [1.62–24.83]**										
**PSC**	*p*										**0.035**	**< 0.001**	**0.004**		**<0.001**	**< 0.001**				
	*OR [95% CI]*										**5.33 [0.95–29.88]**	**12.15 [2.46–60.04]**	**7.25 [1.55–33.77]**							
**Smoking**	*p*				*0*.*007*		*0*.*007*													
	*OR [95% CI]*				*0*.*26 [0*.*09–0*.*74]*		*0*.*26 [0*.*09–0*.*74]*													

Rows corresponding to gender, age at diagnosis, colitis extent and cutaneous manifestations were omitted because statistically significant differences for a given parameter were not obtained; positive associations are indicated in bold and negative associations in italic [*p*-values, odds ratio, and 95% confidence intervals].

ASCA: anti-*Saccharomyces cerevisiae* antibody, EIM: extraintestinal manifestation, LFS: lactoferrin, CUZD1: CUB and zona pellucida-like domains 1, EIMs: extraintestinal manifestations, GP2: glycoprotein 2, P-ANCA: perinuclear anti-neutrophil cytoplasmic antibodies, PSC: primary sclerosing cholangitis

### Significance of serologic antibodies in the risk of unfavorable disease course

In Kaplan-Meier analysis, the presence of certain antibodies was associated with an increased cumulative probability of study-endpoint events compared to the absence of these antibodies (summarized in Table 5 and S1 Table).

**Table 5:** Univariate and multivariate Cox-regression analysis evaluating association between clinical and serologic variables and the study end-point events (ulcerative colitis-related hospitalization and need for immunosuppressant therapy).

.	.	.	UC Related Hospitalisation						Need for Long-Term Immunosupressant Therapy					
.	.	.		.	univariate analysis		mulivariate analysis				univariate analysis		mulivariate analysis	
.	.	n of subject	CP of event (%)*	pLogRank	HR (95% CI)	p-value	HR (95% CI)	p-value	CP of event (%)*	pLogRank	HR (95% CI)	p-value	HR (95% CI)	p-value
Overall population	.	183	32.9	.	.	.	.	.	38.3					
**Clinical factors**	.	.	.	.	.	.	.	.						
Age	A1	10	40.0	0.092	2.0 (0.84–4.77)	0.118			30.0	0.672	1.21 (0.38–3.31)	0.836	0.67 (0.22–2.10)	0.471
	A2	107	34.2	0.402	1.26 (0.73–2.19)	0.405			45.8	**0.048**	1.69 (0.99–2.89)	0.053	1.59 (0.90–2.78)	0.108
	A3	66	29.1						28.3					
Gender	male	82	40.4	**0.016**	**1.82 (1.11–2.98)**	**0.018**	1.36 (0.79–2.34)	0.266	49.9	**0.007**	**1.91 (1.18–3.07)**	**0.008**	1.63 (0.97–2.75)	0.067
	female	101	26.9						29.7					
Maximal disease extent	E1	23	11.7						0.0		**3.09 (2.0–4.77)**	**< 0.001**	**3.15 (1.95–5.10)**	**< 0.001**
	E2	97	28.6	0.094	3.21 (0.77–13.43)	0.111	5.22 (0.71–38.47)	0.105	29.3	**0.013**				
	E3	60	48.4	**0.002**	**6.66 (1.60–27.80)**	**0.009**	**11.67 (1.59–85.56)**	**0.016**	61.7	**< 0.001**				
Smoking	no	164	31.8						36.1					
	yes	19	33.4	0.732	1.14 (0.54–2.40)	0.734			57.9	0.274	1.45 (0.74–2.84)	0.276		
**Serologic** **antibodies**														
Anti-CUZD1 (≈ rPAg1) IgG	no	163	28.8						36.2					
	yes	11	78.6	**0.031**	**2.34 (1.05–5.23)**	**0.038**	2.04 (0.91–4.56)	0.083	78.1	**0.008**	**2.55 (1.25–5.20)**	**0.01**	1.50 (0.68–3.28)	0.316
Anti-CUZD1 (≈ rPAg1) IgA	no	162	29.7						36.8					
	yes	12	63.6	0.068	2.16 (0.91–5.10)	0.077			84.1	**0.005**	**2.78 (1.31–5.89)**	**0.007**	1.51 (0.69–3.32)	0.671
ASCA IgG	no	163	30.3						37.4					
	yes	20	55.0	0.186	1.60 (0.79–3.27)	0.193			46.2	0.313	1.43 (0.71–2.90)	0.315		
ASCA IgA	no	163	31.0						34.7					
	yes	20	50.0	0.158	1.65 (0.81–3.36)	0.165			64.2	**0.003**	**2.43 (1.33–4.46)**	**0.004**	**2.51 (1.33–4.74)**	**0.005**
Number of Abs positivity (Either)	≤2	120	24.3						29.4					
	3≤	49	50.2	**0.016**	**1.93 (1.11–3.35)**	**0.019**	**2.03 (1.16–3.56)**	**0.013**	63.3	**0.0001**	**2.62 (1.57–4.38)**	**0.0002**	**3.19 (1.84–5.53)**	**0.00004**

Rows corresponding to atypical P-ANCA, anti-LFS antibodies, anti-goblet antibodies, anti-GP2 antibodies, and anti-OMP antibodies were omitted because statistically significant differences for a given parameter were not obtained; significant associations are indicated in bold [*p*-values, hazard ratio, and 95% confidence intervals]. Data regarding colectomy is presented in the Supplementary Material (S1 Table).

* CP (cumulative probability) of event (%) corresponds to the median follow-up values

ASCA: anti-*Saccharomyces cerevisiae* antibody, LFS: lactoferrin, CUZD1: CUB and zona pellucida-like domains 1,GP2: glycoprotein 2, P-ANCA: perinuclear anti-neutrophil cytoplasmic antibodies, GCS: glycocorticosteroid

Disease extent: E1: proctitis, E2: left-sided colitis, E3: extensive colitis. Age: A1: ≤ 16 years, A2: 17–40 years, A3: > 40 years

Further analyzing the quantitative associations with unfavorable disease outcomes, we did not find the use of highest quartiles as cut-off values superior compared to the original ones.

Cumulative probability of UC-related hospitalization was significantly higher in anti-CUZD1 IgG (78.6% vs. 28.8%, p_LogRank_ = 0.031), but not in IgA positive cases at 135 months of the follow-up period. In case of the latter antibody, evaluating at higher titer as a cut-off point (≥1:1000; HR_CUZD1IgA_: 1.91 [95% CI: 0.69–5.30]; p = 0.214), similar result was found to that one obtained at lower cut-off value (≥1:10; HR_CUZD1IgA_: 2.16 [95% CI: 0.91–5.10]; p = 0.077).

At the same time, cumulative probability of need for long-term immunosuppressant therapy with azathioprine [AZA] was significantly higher either in anti-CUZD1 IgG (78.1% vs. 36.2%, p_LogRank_ = 0.008) or IgA positive cases (84.1% vs. 36.8%, p_LogRank_ = 0.005) as compared to antibody negative ones. The risk of need for long-term immunosuppressant therapy did not differ according to the extent of anti-CUZD1 IgA antibody positivity (HR_CUZD1IgA_: 2.53 [95% CI: 1.09–5.91]; p = 0.032 for titer of ≥1:1000 and HR_CUZD1IgA_: 2.78 [95% CI: 1.31–5.89]; p = 0.007 for titer of ≥1:10). The presence of IgA as well as IgG type CUZD1 was associated with the need of colectomy, however with only borderline significance without clinically relevant cumulative probability differences (0.0 vs. 5.5%; p_LogRank_ = 0.026 and p_LogRank_ = 0.027, respectively). Comparing higher serum antibody titers (≥1:1000; HR_CUZD1IgA_: 5.58 [95% CI: 1.15–27.04]; p = 0.033) with lower ones (≥1:10; HR_CUZD1IgA_: 5.01 [95% CI: 1.03–24.28]; p = 0.045) carried the same risk.

**Fig 1:**
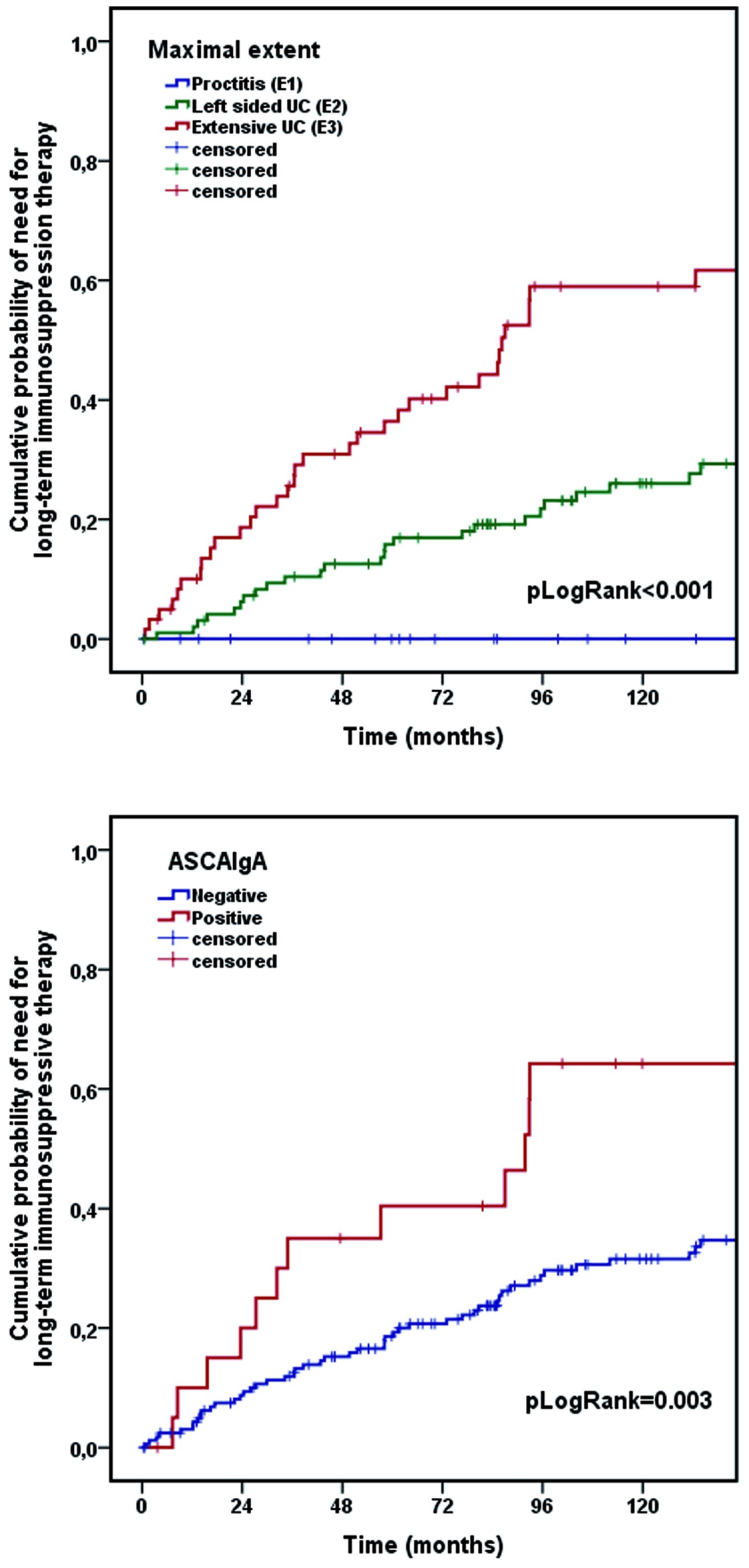
Kaplan–Meier survival plot of need for long-term immunosuppressant therapy with azathioprine in ulcerative colitis during follow-up.

Cumulative probability of UC-related hospitalization did not differ according to IgA or IgG ASCA status. The use of higher cut-off value of IgA type ASCA (≥47 U; HR_ASCAIgA_: 2.34 [95% CI: 0.85–6.50]; p = 0.102) in the analysis yielded similar results to lower titer (≥25 U; HR_ASCAIgA_: 1.65 [95% CI: 0.81–3.36]; p = 0.165). On the contrary, presence of IgA, but not the IgG type ASCA was associated with an increased cumulative probability of the need for long-term immunosuppressant therapy with AZA (64.2% vs. 34.7%, p_LogRank_ = 0.003) (Fig 1). In case of high ASCA IgA antibody titer (≥47 U; HR_ASCAIgA_: 3.55 [95% CI: 1.53–8.25]; p = 0.003) the risk of need for long-term immunosuppressant therapy was similar to those observed at lower positive titer (≥25 U; HR_ASCAIgA_: 2.43 [95% CI: 1.33–4.46]; p = 0.004). However only the presence of IgG type ASCA was moderately associated with need of colectomy (13.6% vs. 3.8%, p_LogRank_ = 0.014).

As for IgA or IgG type atypical P-ANCA, anti-LFS, GAB or IgA type anti-OMP antibodies, no differences between antibody positive and negative patients were observed in terms of either the study-endpoint events (Table 5).

#### Covariates

Analysis of clinical factors associated with UC-related hospitalization and requirement for long-term immunosuppressant therapy with azathioprine using Kaplan-Meier and univariate Cox-regression analysis is shown in Table 5. Colitis extent (Fig 1) and male gender but neither age of onset nor smoking habits were significantly associated with these study endpoints.

None of the clinical factors were significantly associated with need for colectomy (S1 Table).

Lastly, development of extensive disease was also considered as an unfavorable outcome. In patient presenting with disease location E1 or E2 (n = 134) none of the examined serologic antibodies were associated with a change to a more extended disease (E3 according to Montreal classification) (S2 Table).

#### Multivariate analysis

Cox-regression analysis and the backward elimination procedure, taking serologic antibodies and all clinical covariates into account, indicated that out of the serologic markers, the presence of IgA type ASCA was independently associated with the higher risk of need for long-term immunosuppressant therapy with AZA (HR: 2.51, 95%CI: 1.33–4.74, p = 0.005). None of the serologic antibodies were independently associated with the higher risk of the UC-related hospitalization (Table 5).

From the clinical parameters, extensive colitis was associated with a higher risk of UC-related hospitalization (HR: 11.67, 95%CI: 1.59–85.56, p = 0.016), and the need for long-term immunosuppressant therapy with AZA (HR: 3.15, 95%CI: 1.95–5.10, p<0.001) (Table 5).

Evaluation of multiple positivity for different antibodies was performed; co-existence of three or more different types of antibodies was associated with UC-related hospitalization along with long-term immunosuppressant therapy but not associated with development of extensive disease or need for colectomy in univariate and multivariate time dependent analysis as well. These result appeared to be superior to single antibody positivity in these unfavorable disease outcomes (Table 5).

## Discussion

In the present study, we investigated the clinical importance of an extensive panel of serologic antibodies comprising both classic and newly discovered auto- and anti-microbial antibodies in the prediction of the long-term disease course in adult UC patients. To our knowledge, this is the largest prospective referral cohort to date, which has been examined by such a wide range of serologic antibodies.

In our cohort, the seropositivity rate of classic serologic antibodies, namely atypical P-ANCA and ASCA, and also anti-OMP antibody corresponds to those previously reported in UC (45–82%, 5–15%, and 20–24%, respectively) [1]. It should be noted, however, that IgA type anti-OMP antibody examined in the present study is clearly different from anti-OmpC. Similar prevalence rate of anti-OmpC (5–28%) [1] and resemblance in nomenclature sometimes causes confusion in the literature. Anti-OMP antibody is directed against multiple bacterial proteins derived from two species of intestinal bacteria (one gram positive and one gram negative). Neither bacteria are from the phylum proteobacteria, of which *Escherichia coli* is a member. At the same time, anti-OmpC antibody is specifically directed to the outer membrane protein C transport protein of *Escherichia coli*. Fewer data are available regarding the prevalence of target specific PABs (anti-GP2 and anti-CUZD1) in patients with UC. In the largest study assessing UC patients (n = 136), both the anti-GP2 and the anti-CUZD1 seropositivity rates were low, 2.9% and 5.9%, respectively, similar to our findings [27].

Prognostic value of serologic antibodies relies on documentation of their stability over time. Accordingly, in the present study we extensively assessed the long-term stability of various antibodies. We found the status of serologic antibodies was not associated with actual disease activity, and positivity rates were stable over time. Most studies in UC that have measured antibodies during active and inactive disease have shown no correlation between P-ANCA and disease severity [8]. Regarding antibody stability, in a previous study of *Vecchi et al*.[12] atypical p-ANCA IgG status remained constant over time (50.8 month time period) when evaluated at more than one time point in a small cohort of UC patients (n = 40). Change in antibody status occurred in 25% of patients, similar to our findings. In our cohort changes of IgG subtype of atypical p-ANCA was 14.4%, while IgA subtype was 23.2%. ASCA and other serologic antibodies showed even lower variation (≤ 10% of cases). This is consistent with previous data provided by *Rieder et al*. [28]. Anti-glycan antibody (such as ASCA) status remained unchanged from the status determined at the initial sample procurement in the vast majority of UC and CD patients. The median time between sample procurements, however, was relatively short (6.2 months).

Reports regarding association of serologic markers with long-term disease course in UC have generally been restricted to the evaluation of atypical P-ANCA and ASCA. Newly identified antibodies have not been well studied in this clinical setting. Possible differences according to antibody subtypes (IgA or IgG type) have also not been within the scope of these studies. Our previous findings that IgA, but not IgG types of PAbs, were associated with complicated disease course in patients with CD support this latter approach [15]. In the present study we aimed to fill these gaps.

In previous longitudinal clinical studies, association between serologic antibodies and adverse disease outcome yielded somewhat discordant results, for various reasons. From the clinical point of view, unfavorable disease outcome—beyond colectomy—was not defined in a unified manner in these studies. In addition, study populations were different as well as regarding the sample size, study design (referral or population-based patient cohort) or follow-up time. It is known that the proportion of IBD patients developing an unfavorable disease outcome could be significantly different in referral and population-based cohorts [29]. Likewise reported prevalences of serologic antibodies are lower in population-based cohorts [11].

In the present study, four primary end-points were selected to define unfavorable disease outcome in UC: development of extensive disease, need for colectomy, requirement for one or more UC-related hospitalization due to disease activity and need for long-term immunosuppressant therapy with AZA.

A change to a more extended disease (E3 according to Montreal classification) can be considered as an unfavorable disease outcome worth to evaluate, however only limited data is available in the literature regarding proximal disease progression over time, as well as the related factors, especially serologic markers having an impact on this outcome. The majority of studies were conducted on this matter more than 20 years ago [30–34].Rate of disease extent progression reported previously varies from 15% to 53% depending on disease duration at the end of follow-up time (5, 10 and 25 years) [30–43]. Until now, the most thorough over time extent evaluation was presented in a Swiss IBD Cohort Study (n = 918), where 9.48% of UC patients (E1 or E2) developed E3 disease during follow up (median time: 9 years) [41]. We found similar progression rate to an extensive disease (n = 134) in our UC patients (12.7%) with similar median follow up time (8.6 years). The strength of our study is that we analyzed for the first time, whether the presence or absence of the classic and novel antibodies are associated with a shorter time to development of an extensive disease, however we failed to prove any significant association. Although, the lack of prognostic potential of these antibodies in this particular outcome should be interpreted cautiously due to low event and patient numbers in antibody positive groups. Former small-scale referral cohort studies demonstrated [12, 44, 45] that the presence of P-ANCA was associated with the need for colectomy in UC. However, more recent large-scale studies, either in the population-based [11, 13] or referral [9, 10, 46] cohorts, have not been able to confirm these early reports. Two population-based studies (Norwegian IBSEN study [13], n = 357 and EC-IBD multicenter study[11], n = 432) did not demonstrate increased risk of colectomy in the presence of P-ANCA or ASCA seropositivity [13]. Two additional referral cohort studies from Canada [9, 10] further confirmed the lack of association between serologic antibodies and need for colectomy. Beyond P-ANCA and ASCA seropositivity, other serologic antibodies, such as anti-OmpC or CBir1 [9], were also not associated with the risk of colectomy. Only one single study [14] found that anti-OmpC positivity was associated to the requirement for colectomy. In the present referral cohort study, we also did not find clinically relevant associations between the requirement for colectomy and the presence of either the classic or the newly identified serologic antibodies, including anti-OMP. The anti-OMP assay used in current study is significantly different from anti-OmpC assay, as previously mentioned.

Concerning UC-related hospitalization as an unfavorable diseases outcome, no significant association was found with P-ANCA and ASCA seropositivity in a recent large-scale referral cohort study of *Kevans et al*.[9](n = 230). Colitis extent was the single variable of the clinical factors that associated with the study endpoint (HR 2.7, 95%CI: 1.5–4.6, p = 0.006). In agreement with that study, only the disease extent, and not any of the serologic antibodies, was able to predict UC-related hospitalization (HR 11.7, 95%CI: 1.6–85.6, p = 0.016) in our cohort.

Requirement for, or response to, certain medical therapies as an adverse outcome in UC was also evaluated in former studies. Mainly corticosteroid or biological therapy was assessed either individually [9] or in combination as components of prognostic profile groups describing disease severity [10]. The need for more intense treatment with AZA was assessed in a single study of *Soleberg et al*.[13]). P-ANCA positive patients had about 4-fold higher risk of receiving AZA treatment during follow up (OR: 4.14, 95%CI: 1.73–9.82, *p = 0*.005). However, in our study, ASCA, and not the P-ANCA seropositivity was associated with a more active course of UC, as there was a significant relationship between presence of ASCA and the overall use of AZA. Interestingly, only IgA, but not IgG type of antibody showed this link. Gut mucosal immune system plays a central role in the IgA antibody formation, and this may at least partly reflect an immune response against an overwhelming microbial challenge. In addition, IgA type autoantibodies are considered as a sign of immunological response to enteric antigens in other diseases associated with enhanced bacterial translocation. Our group reported that IgA type antibodies have a pivotal role in the development of disease-specific complications compared with the IgG antibody subtype [47]. Remarkably, in the present cohort the occurrence of IgA type target specific PAbs but not IgG type was significantly higher in those patients with concomitant PSC. The same association was reported previously [15] in a cohort of our CD patients. That was confirmed later by *Michaels et al*. [27] in UC and CD as well. These findings might serve as an additional hint towards the importance of gut mucosal immune system dysfunction in the development of hepatobiliary manifestations [48].

Based on the experience gained from previous serological studies in IBD [1] including those performed by us as well, we know that an increasing number or magnitude of seropositivity can yield higher association with disease complications than single markers. In the present study, however we were not able to confirm that the use of highest quartiles as cut-off values were superior compared to the original ones. Although, we have to highlight that the lack of associations regarding highest antibody titers can be the result of a very limited number of patients belonging to these categories. Distinctly, multiple seropositivity, namely the co-existence of three or more different types of antibodies, results proved to be superior compared to single antibody positivity regarding certain outcomes, such as UC-related hospitalization and need for long-term immunosuppressant therapy.

This study has some limitations: (1) our hospital is a regional referral center for IBD patients introducing a selection bias; (2) relatively small number of subjects underwent colectomy but it is in accordance with previous reports from Eastern Europe [49]; thus any lack of significant association could also be explained by insufficient statistical power (type 2 error); (3) the wide range of seropositivity of the examined antibody panel (9–73%) did not make possible an equally powered evaluation in case of each certain markers and warrants further validation in larger patient cohort. (4) our patient cohort is followed prospectively and the database is updated regularly for that concern. Serum sampling, however, occurred later in subject’s disease course rather than at or soon after diagnosis. Median disease duration was 4 years at serum drawing which is a significantly shorter interval than in previous studies. At the same time, sufficient prospective follow-up (median, 11 years) was available after sampling. Seventy-six percent of our patients had at least 5 years of follow-up which is the period suggested by *Silverberg et al*.[10] that is required for evaluation of long-term outcomes. Based on these and the stability data of the present study, we believe that our serologic findings provide reliable prognostic information for the whole disease course of UC, including near the time of the diagnosis as well.

In conclusion, consistent with the majority of previous reports, we have shown that presence of atypical P-ANCA is not associated with unfavorable disease outcome in UC. We did not demonstrate any association of newly identified serologic antibodies with the unfavorable disease outcome. We demonstrated, however, a novel association between the presence of IgA, but not the IgG type ASCA and requirement for long-term immunosuppressant therapy with AZA. Assessment of serologic antibody subtypes may prove to be an important novel parameter. Further studies are now needed to validate and extend these results.

## Supporting information

S1 Table Univariate and multivariate Cox-regression analysis evaluating association between clinical and serologic variables and the omitted study end-point colectomy. (DOCX)

S2 Table Summary of Kaplan-Meier survival analysis forthe probability of the development of extensive disease (E3) in UC patients. (DOCX)

S1 File Post-hoc power analysis of antibody seropositivity and poor disease outcome (UC-related hospitalization and need for long-term immunosuppressant therapy). (DOCX)

## Abbreviations

ANCA: anti-neutrophil cytoplasmic antibodies
ASCA: anti-*Saccharomyces cerevisiae* antibody
AZA: azathioprine
BT: bacterial translocation
CD: Crohn’s disease
CI: confidence interval
CUZD1: CUB and zona pellucida-like domains 1
ELISA: enzyme-linked immunosorbent assay
GP-2: glycoprotein 2
HR: hazards ratio
IBD: inflammatory bowel diseases
Ig: immunoglobulin
IIFT: indirect immunofluorescence test
IQR: inter quartile range
LFS: lactoferrin
OR: odd ratio
PAB: pancreatic antibody
P-ANCA: perinuclear anti-neutrophil cytoplasmic antibodies
PSC: primary sclerosing cholangitis
TNF: tumor necrosis factor
UC: ulcerative colitis
