# Supplementary material for: Significance of serological markers in the disease course of ulcerative colitis in a prospective clinical cohort of patients
Source: PLoS One. 2018 Mar 28;13(3):e0194166. doi: 10.1371/journal.pone.0194166 (PMC5874003; doi:10.1371/journal.pone.0194166)
Supplement: S1 Table — (DOCX) [file pone.0194166.s001.docx]

**S1 Table** . **Univariate and multivariate Cox-regression analysis evaluating association between clinical and serologic variables and the omitted study end-point colectomy.**

| . | . | . | **Need for colectomy** | | | | | |
| --- | --- | --- | --- | --- | --- | --- | --- | --- |
| . | . | . |  | . | univariate analysis | | mulivariate analysis | |
| . | . | n of  subject | CP of  event  (%)* | pLogRank | HR (95% CI) | p-value | HR (95% CI) | p-value |
| Overall population | . | 183 | 32.9 | . | . | . | . | . |
| **Clinical factors** | . | . | . | . | . | . | . | . |
| Age | A1 | 10 |  |  |  | 0.986 |  |  |
|  | A2 | 107 | 6.5 | 0.196 | 2.77 (0.60-12.83) | 0.192 |  |  |
|  | A3 | 66 | 3.1 | 0.579 |  |  |  |  |
| Gender | male | 82 | 5.4 | 0.755 | 1.21 (0.37-3.97) | 0.756 |  |  |
|  | female | 101 | 4.4 |  |  |  |  |  |
| Maximal  disease extent | E1 | 23 | 0.0 |  |  |  |  |  |
|  | E2 | 97 | 3.3 | 0.372 | 22190.73 (0-5.9E) | 0.949 |  |  |
|  | E3 | 60 | 8.8 | 0.230 | 34125.312 (0-90.7E) | 0.947 |  |  |
| Smoking | no | 164 | 4.3 |  | 1.77 (0.38-8.23) | 0.466 |  |  |
|  | yes | 19 | 9.1 | 0.459 | 0 (0-) | 0.990 |  |  |
| **Serologic**  **antibodies** |  |  |  |  |  |  |  |  |
| Anti-CUZD1  (≈ rPAg1) IgG | no | 163 | 5.5 |  |  |  |  |  |
|  | yes | 11 | 0 | **0.027** | **5.02 (1.02-24.69)** | **0.047** |  |  |
| Anti-CUZD1  (≈ rPAg1) IgA | no | 162 | 5.5 |  |  |  |  |  |
|  | yes | 12 | 0 | **0.026** | **5.01 (1.03-24.28)** | **0.045** |  |  |
| ASCA IgG | no | 163 | 3.8 |  |  |  |  |  |
|  | yes | 20 | 13.6 | **0.014** | **4.67 (1.2-18.23)** | **0.027** |  |  |
| ASCA IgA | no | 163 | 4.7 |  |  |  |  |  |
|  | yes | 20 | 5 | 0.173 | 2.82 (0.59-13.35) | 0.192 |  |  |
| Number of Abs positivity (Either) | ≤2 | 120 | 5 |  |  |  |  |  |
|  | 3≤ | 49 | 5.6 | 0.140 | 2.58 (0.70-9.54) | 0.154 |  |  |

Rows corresponding to atypical P-ANCA, anti-LFS antibodies, anti-goblet antibodies, anti-GP2 antibodies, and anti-OMP antibodies were omitted because statistically significant differences for a given parameter were not obtained; significant associations are indicated in bold [*p*-values, hazard ratio, and 95% confidence intervals].

* CP (cumulative probability) of event (%) corresponds to the median follow-up values

ASCA: anti-*Saccharomyces cerevisiae* antibody, LFS: lactoferrin, CUZD1: CUB and zona pellucida-like domains 1, GP2: glycoprotein 2, P-ANCA: perinuclear anti-neutrophil cytoplasmic antibodies, GCS: glycocorticosteroid

Disease extent: E1: proctitis, E2: left-sided colitis, E3: extensive colitis. Age: A1: ≤ 16 years, A2: 17-40 years , A3: > 40 years
