# Supplementary material for: Significance of serological markers in the disease course of ulcerative colitis in a prospective clinical cohort of patients
Source: PLoS One. 2018 Mar 28;13(3):e0194166. doi: 10.1371/journal.pone.0194166 (PMC5874003; doi:10.1371/journal.pone.0194166)
Supplement: S2 Table — (DOCX) [file pone.0194166.s002.docx]

**S2 Table. Summary of Kaplan-Meier survival analysis for the probability of the development of extensive disease (E3) in UC patients**

| **Kaplan-Meier survival analysis** | **Development of extensive disease (E3)** |
| --- | --- |
| **Serologic antibodies** | **long rank p** |
| Atypical P-ANCA IgG | 0.395 |
| Atypical P-ANCA IgA | 0.425 |
| Anti-LFS IgG | 0.117 |
| Anti-LFS IgA | 0.424 |
| Anti-goblet cells IgG | 0.886 |
| Anti-goblet cells IgA | 0.145 |
| Anti-CUZD1 (≈ rPAg1) IgG | 0.446 |
| Anti-CUZD1 (≈ rPAg1) IgA | 0.281 |
| Anti-GP2 (≈ rPAg2) IgG |  |
| Anti-GP2 (≈ rPAg2) IgA |  |
| ASCA IgG | 0.280 |
| ASCA IgA | 0.172 |
| Anti-OMP IgA | 0.355 |
| Number of Abs positivity (Either) (3≤) | 0.169 |
