# Supplementary material for: Significance of serological markers in the disease course of ulcerative colitis in a prospective clinical cohort of patients
Source: PLoS One. 2018 Mar 28;13(3):e0194166. doi: 10.1371/journal.pone.0194166 (PMC5874003; doi:10.1371/journal.pone.0194166)
Supplement: S1 File — (DOCX) [file pone.0194166.s003.docx]

**S1 File**

**Post-hoc power analysis of antibody seropositivity and poor disease outcome (UC-related hospitalization and need for long term immunosuppressant therapy)**

Considering the range of seropositivity of 9-73%, a **post-hoc power analysis was performed** in Stata (v13.0), in which the following factors were considered:

- Sample size: **174**
- Antibody positivity range suggested by the Reviewer taken into consideration (highest [atypical pANCA IgA and/or IgG]: **73%**, lowest [anti-CUZD1 IgA and/or IgG]: **9%**)
- Cumulative probability* of event in control group:
- UC related hospitalization: **54.3%** and **50.7%**
- Need for long term immunosuppressant therapy: **42.5%** and **51.8%**

for pANCA and anti-CUZD1 IgA and/or IgG as risk factors, respectively.

- Clinically relevant hazard ratio: **2**
- Type I error: **0.05, two-sided**

1. **UC-related hospitalization - Results with a constant hazard ratio (HR), using 2 as a clinically relevant HR**

| Risk variant | Absent/  present | Event | Probability of **UC-related hospitalization** in groups risk variant absent | Probability of **UC-related hospitalization** in groups risk variant present | HR | Alpha | Power |
| --- | --- | --- | --- | --- | --- | --- | --- |
| ***Atypical pANCA*** *IgA and/or IgG* | 44/134 | 58 | 0.543 | 0.518 | 2 | 0.05 | **0.821** |
| ***Anti-CUZD1*** *IgA and/or IgG* | 16/158 | 58 | 0.507 | 0.670 | 2 | 0.05 | **0.712** |

1. **Need for long term immunosuppressant therapy - Results with constant hazard ratio (HR), using 2 as a clinically relevant HR**

| Risk variant | Absent/  present | Event | Probability of **need for long term immunosuppressant therapy** in groups risk variant absent | Probability of **need for long term immunosuppressant therapy** in groups risk variant present | HR | Alpha | Power |
| --- | --- | --- | --- | --- | --- | --- | --- |
| ***Atypical pANCA*** *IgA and/or IgG* | 44/130 | 66 | 0.425 | 0.598 | 2 | 0.05 | **0.748** |
| ***Anti-CUZD1*** *IgA and/or IgG* | 158/16 | 66 | 0.518 | 1.000 | 2 | 0.05 | **0.721** |

We found that the **power associated** with atypical pANCA and anti-CUZD1 (IgA and/or IgG) for detecting a difference in UC-related hospitalization were **82.1%** and **71.2%**, respectively. In case of need for long term immunosuppressive therapy these were **74.8 %** and **72.1%**. These power analysis results show that our study is slightly underpowered regarding the evaluated outcomes. At the same time the strength of our study was that the median follow up time was fairly long (135 months), which made the evaluation of the proposed associations possible in long term.
